# Where myth and archaeology meet: Discovering the Gorgon Medusa’s Lair

**DOI:** 10.1371/journal.pone.0249606

**Published:** 2021-04-01

**Authors:** Clive Finlayson, Jose Maria Gutierrez Lopez, M. Cristina Reinoso del Rio, Antonio M. Saez Romero, Francisco Giles Guzman, Geraldine Finlayson, Francisco Giles Pacheco, David Abulafia, Stewart Finlayson, Richard P. Jennings, Joaquin Rodriguez Vidal

**Affiliations:** 1 The Gibraltar National Museum, Gibraltar, Spain; 2 The University of Gibraltar, Gibraltar, Spain; 3 Department of Anthropology, University of Toronto, Toronto, Canada; 4 School of Biological and Environmental Sciences, Liverpool John Moores University, Liverpool, United Kingdom; 5 Museo Histórico Municipal de Villamartín, Cádiz, Spain; 6 Department of Prehistory and Archaeology, University of Seville, Seville, Spain; 7 Gonville and Caius College, Cambridge University, Cambridge, United Kingdom; 8 Department of Life Sciences, Anglia Ruskin University, Cambridge, United Kingdom; 9 Department of Earth Sciences and CIPHCN-Heritage Centre, University of Huelva, Huelva, Spain; Universidad de Cádiz, Facultad de Ciencias del Mar y Ambientales, SPAIN

## Abstract

Here we report the discovery of ceramic fragments that form part of a Gorgoneion, a ceramic image representation of the Gorgon Medusa. The fragments were found in a deep part of Gorham’s Cave, well known to ancient mariners as a natural shrine, between the 8^th^ and 2^nd^ century BCE. We discuss the context of this discovery, both within the inner topography of the cave itself, and also the broader geographical context. The discovery is situated at the extreme western end of the Mediterranean Sea, where it meets the Atlantic Ocean. The location was known to ancient mariners as the northern Pillar of Herakles, which marked the end of the known world. We relate the discovery, and its geographical and chronological context, to Greek legends that situated the lair of the Gorgon sisters at a location which coincides with the physical attributes and geographical position of Gorham’s Cave. We thus provide, uniquely, a geographical and archaeological context to the myth of Perseus and the slaying of the Gorgon Medusa.

## Introduction

The quest for sites and artefacts of classical mythology was the hallmark of archaeology at the end of the nineteenth century. Schliemann’s excavations in search of Troy and Evans’ work at Knossos are among the best known [1,2]. The purported discoveries of King Priam’s treasure or the mask of Agamemnon are prime examples of attempts to link material culture to classical stories [3,4]. These missions and their controversial results constituted a major part of early archaeology and have no parallel today, with the possible exception of the renewed search for Atlantis at the start of the 21^st^ Century [5]. Establishing clear links between material culture, sites and ancient stories remains as elusive today as it was over a hundred years ago. Here we report the discovery of fragments of a ceramic image of the Gorgon Medusa from Gorham’s Cave, Gibraltar. These are the only such remains, representing Medusa, known from inside a cave. The location of the discovery, deep in a cave situated at the base of one of the Pillars of Herakles which marked the end of the world, supports the view that the ancient mariners regarded this to have been the home of the Gorgons.

## Site and context

**Fig 1:**
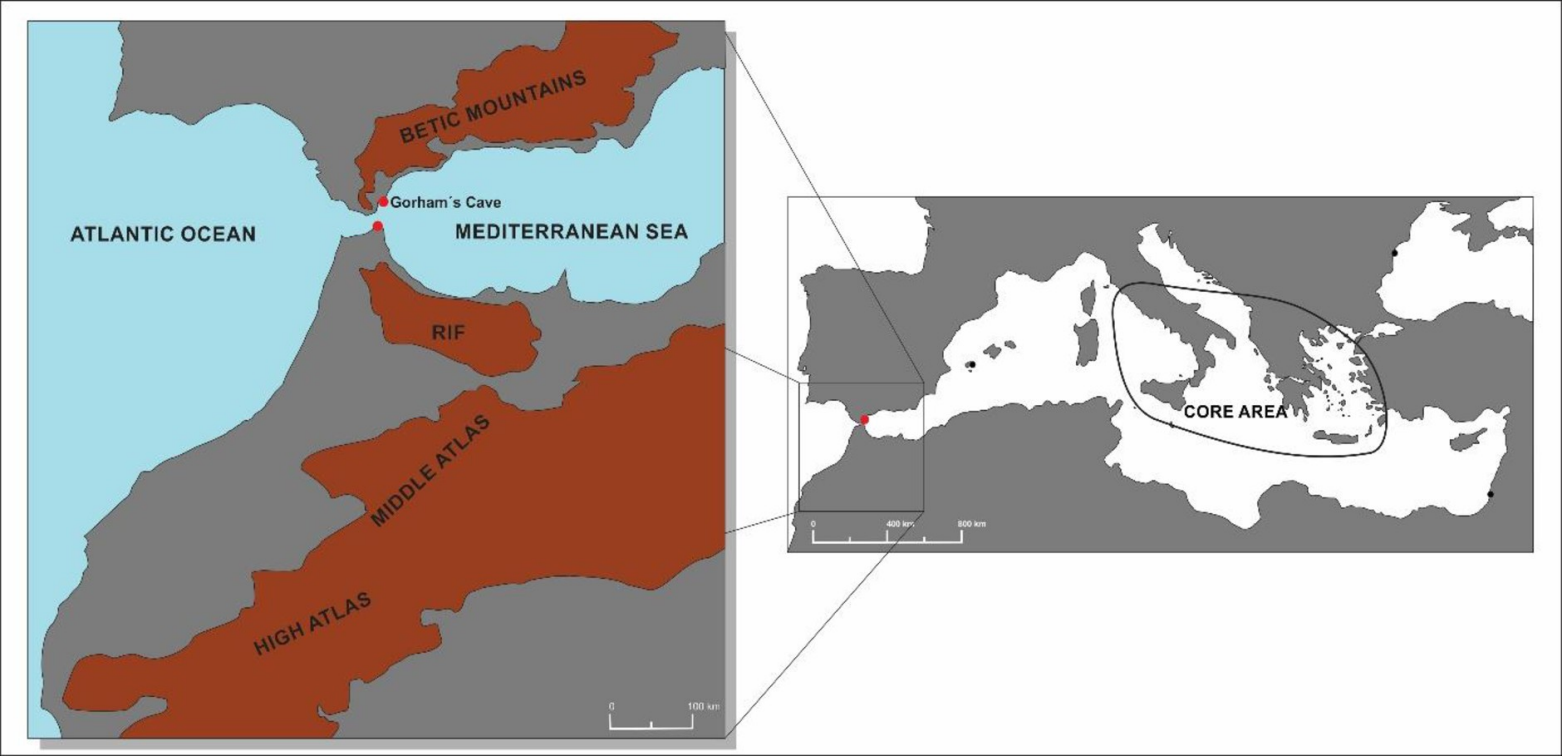
Map of the Mediterranean Sea showing the core area where Gorgoneia have been found. Exceptions outside the core area are in the Black Sea, Israel and Ibiza. The location of Gibraltar is indicated by a red dot. Details of individual sites are at S2 File. Inset shows the region of the Strait of Gibraltar, showing the location of Gorham’s Cave. The two Pillars of Herakles are marked by red dots, Gibraltar (Mons Calpe) being the northern Pillar. The location of the Atlas Mountains is indicated.

The Pillars of Herakles (Fig 1) were a major geographical landmark for mariners during the first millennium BCE [6]. Pindar, in the sixth century BCE recommended mariners not to sail beyond the Pillars [7]. They were not just the markers of the end of the known world, they were also invested in symbolic significance, immersed in the world of myth for the Mediterranean civilizations of the time. For the Greeks, the unknown and alien world of the ocean beyond the columns provided an ideal frame for situating lands and mythological events that since the time of Homer had been considered to reside at the extreme west of their world. These geographical peculiarities, along with the dangers of navigating the unpredictable Strait of Gibraltar and the topographically abrupt and immense nature of the northern Pillar, the Rock of Gibraltar itself, would have been decisive in the sacralisation of the most notable natural features of this part of the world [8]. The massive, cathedral-like, Gorham’s Cave which is situated at the base of the Rock on its eastern side facing the Mediterranean Sea, appears to have been the most important among these. Archaeological work in this cave has uncovered a clear signal, in the form of a rich material culture of votive offerings left inside by ancient peoples, of its role as a place of worship at the very end of the world [8–11].

## Results

Twelve ceramic fragments that had once formed part of a Gorgoneion, a terracotta depicting the face of the Gorgon Medusa [12], were retrieved during excavations of an archaeological level at Gorham’s Cave, known to have been visited by people between the first half of the eighth century BCE and the mid-second century BCE [8].

**Fig 2:**
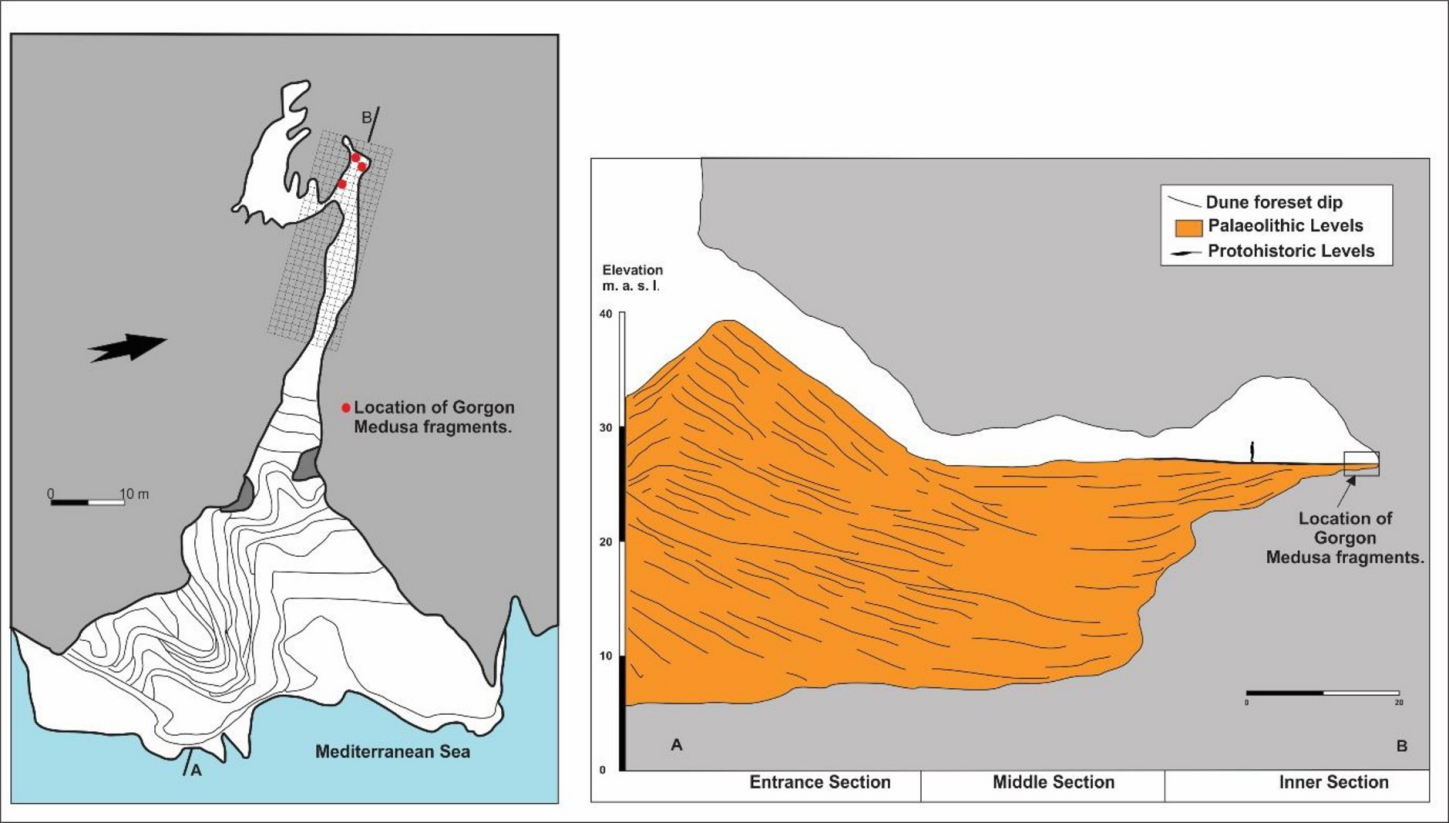
Map and section of Gorham’s Cave showing location of the Gorgoneion fragments in the deepest part of the cave where it connects with the entrance to a deep and narrow chamber. The section shows the dune covering the entrance to the cave as it would have appeared at the time that the cave was being used as a coastal shrine.

**Fig 3:**
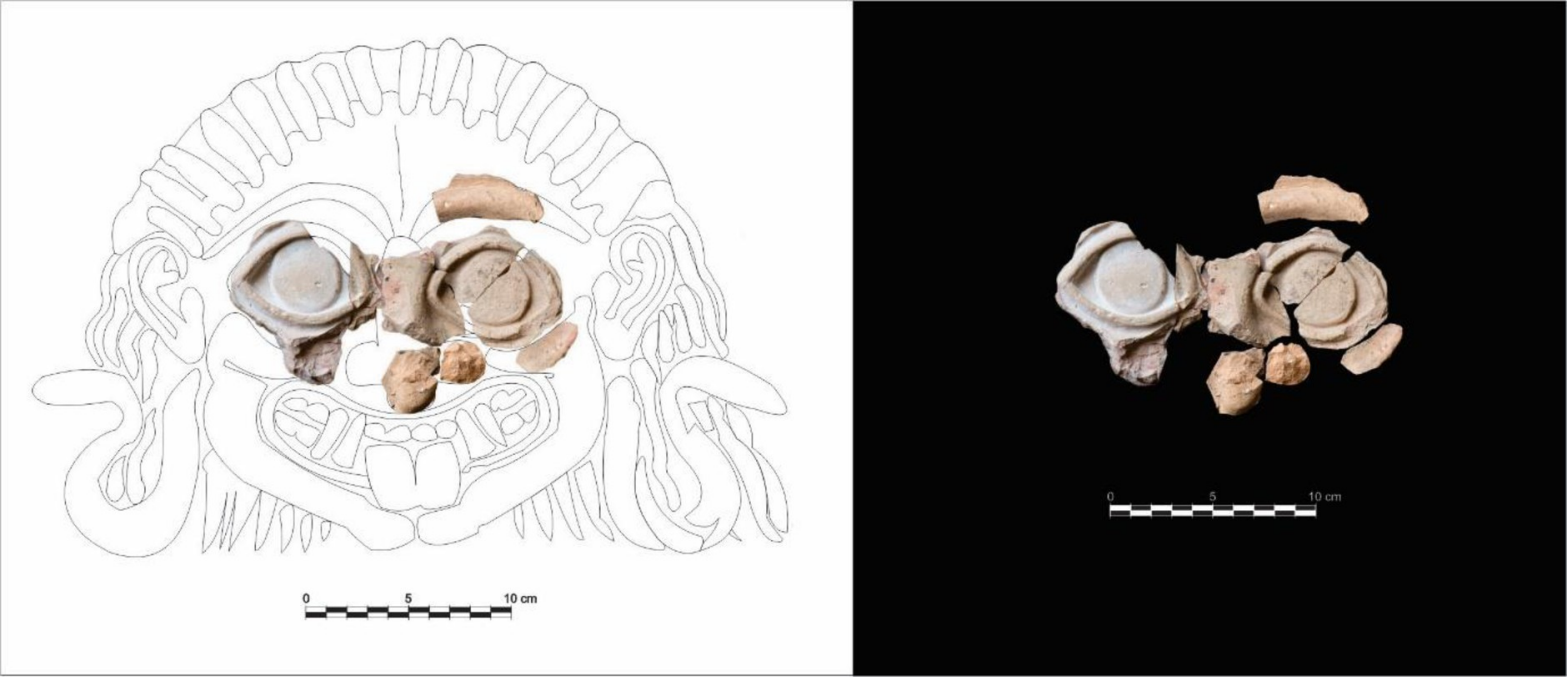
**Fragments recovered from Gorham’s Cave (right) with a reconstruction of the complete Gorgoneion.** See also S1 File. Specimen IDs: GOR/’98/B3/52a; GOR/’98/B3/52b; GOR/’98/B3/52c; GOR/’98/B3/52d; GOR/’98/B3/52e; GOR/’98/B3/52f; GOR/’98/B3/52g; GOR/’98/B3/52h; GORHAM’S C 1958 GA7(a); GORHAM’S C 1958 GA7(b); GOR99/alpha2/N I/140; GOR99/alpha alpha2/N I/36. Stored at The Gibraltar National Museum, Gibraltar. All necessary permits were obtained for the described study, which complied with all relevant regulations.

The ceramic fragments were found in the deepest part of Gorham’s Cave (S1 File), in a location where there is a small opening that leads to a 35-metre long unexcavated chamber which is only accessible by crawling through very tight spaces (Fig 2). The fragments consist of both eyes, part of the nose, cheeks and the left eyebrow (Fig 3). The large size of the pieces suggest that this would have been a large artefact, with a maximum width of 36.5 cm and a maximum height of 26.5 cm. A literature survey has revealed a total of 91 published Gorgoneia (S2 File). With the exception of one, from a necropolis in Ibiza, and another from a temple on the coast of the Black Sea, the rest are all from the central and eastern Mediterranean (Fig 1). None are from cave contexts, making the Gorham’s Cave piece exceptional. Stylistically, the Gorham’s Cave Gorgoneion corresponds to the sixth century BCE and is of the horrifying and repulsive type; after this time such items gradually take on a more human aspect.

## Discussion

Access to Gorham’s Cave at the time would have been from the Mediterranean Sea and would have required boats. Until now the interpretation, based on a combination of material culture excavated, and the known presence of these people in the area at the time, has been that they were Phoenician and later Carthaginian mariners. Recent analyses have shown that the material culture found in this level has a broader international character, including Egyptian, Greek, Sardinian and Tartessian (i.e. southern Iberian) items [11]. It is possible that such items were traded by the Phoenicians but the occurrence of other mariners in the cave cannot be discarded. The presence of a Gorgoneion, similarly, need not reflect a direct Greek introduction as such items have occasionally been found in Phoenician contexts [13–15], although in the case of the Gorham’s Cave Gorgoneion, its style and fabric leaves little doubt as to its Greek origins. Furthermore, recent finds are indicating a Greek participation in cult activities in Phoenician-Tartessian sanctuaries along the Atlantic coast of south-western Iberia, beyond the Strait of Gibraltar itself [16]. There is no other example of this type of Gorgoneion, of similar size or chronology, in the western Mediterranean; it is therefore an exceptional find that cannot be clearly linked to any Phoenician deity and implicates the Greeks instead. We do not overlook however, the fact that deities from other contemporary Mediterranean cultures may have had a degree of equivalence among the early Mediterranean mariners. In particular, part of a figurine of Egyptian Bes, (a more benevolent, ugly-bearded faced creature), whose figure was used profusely among Egyptians and Phoenicians for protection, with a crossover in the Greek versions of the Gorgon that are male-bearded, has been described from Gorham’s Cave [8].

The Gorgons were three sisters, the most evil of which–Medusa–was slain by Perseus. There are clear indications from several classical sources that the events surrounding this significant moment in Greek mythology were portrayed as having taken place in the area of the Strait of Gibraltar: (a) The Titan Atlas ruled over a kingdom in the far west, beyond the Pillars of Herakles; (b) Perseus stole the single eye of the Graeae, the Gorgons’ sisters, in order to find the Stygian nymphs in whose care was the dark helmet of invisibility which belonged to Hades and which he needed to complete the task of killing the Medusa. Perseus sought the Graeae on their thrones at the foot of Mount Atlas; (c) Perseus escaped after beheading Medusa and sought refuge near the palace of Atlas who refused to offer hospitality. Perseus showed Medusa’s head to Atlas and transformed him into a mountain; (d) the three Gorgons lived close to a far-western orchard, the Garden of the Hesperides [see below] [17].

The poet Hesiod (late 8^th^-early 7^th^ century BCE) is the principal early author referring to the Perseus myth in his *Theogony* and clearly situates events in the Strait of Gibraltar area: (a) he places the Gorgons in the Garden of the Hesperides, at the limits of the Atlantic Ocean—“the Gorgons who dwell beyond glorious Ocean at the edge towards the night, where the clear-voiced Hesperides are, Sthenno and Euryale, and Medusa who suffered woes”; and (b) “When Perseus cut her head off from her neck, great Chrysaor and the horse Pegasus sprang forth; the latter received his name from being born beside the waters of Ocean… Chrysaor… begot three-headed Geryoneus, who was slain by Herakles’ force… after he crossed over the strait of Ocean and killed Orthus and the cowherd Eurytion in the murky stable beyond glorious Ocean.” [18].

Remoulding older material, Ovid (43 BCE-17/18 CE) places the sisters who shared an eye in a cave beneath Mount Atlas. In his long poem *Metamorphosis*, Ovid gives us further clues regarding the geographical location of Perseus’ story: “Now tell us, pray, O Perseus, by what wondrous valour, by what acts, you won the Gorgon’s snaky head. The hero, answering, told how beneath cold Atlas there was a place safe under the protection of the rocky mass. At the entrance to this place two sisters dwelt, both daughters of old Phorcys [sic], who shared one eye between them…” [19].

The most direct reference situating Medusa in the Rock of Gibraltar itself comes from the Roman poet Silius Italicus (~28–103 CE): “Down went huge Phocrys, who came from the caves of Calpe [the Latin name for Gibraltar], sacred to Hercules; on his shield was engraved the Gorgon’s head; for that cruel goddess derived her birth and beginning from Calpe.” [20].

In her essence Medusa is a head and she acquires her potency, which resides in the head, only after it has been severed from the body. She is in effect a mask with a body appended [12]. Such artefacts were part of a religion of terror among the Greeks and were a way of frightening strangers from secret or mysterious places [17]. In the case of Gorham’s Cave, the location of the find suggests that it may have been the deep inner chamber that it was protecting.

**Fig 4:**
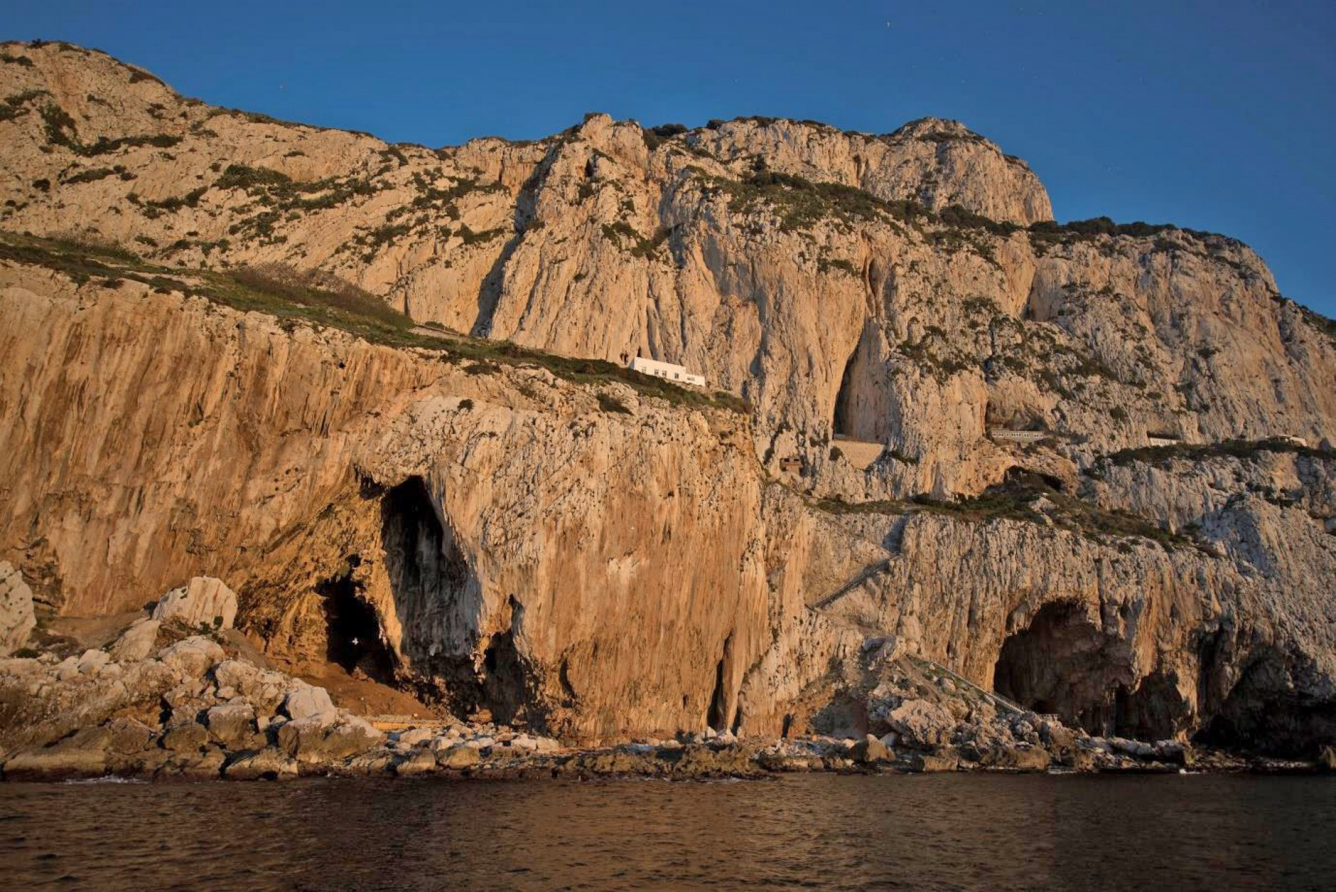
Gorham’s Cave at the base of Mons Calpe (Gibraltar) from the sea today.

The Perseus story is the product of a long oral tradition and is thought to have reached its current form between 700 and 650 BCE, a time frame which broadly corresponds to Hesiod’s *Theogony*, when the Medusa story is fully developed [21], and with the date of the Gorham’s Cave Gorgoneion. The inner chamber at Gorham’s Cave clearly had a special significance to the people who stopped there, one associated with mystery and sea travel, and protected by the image of Medusa in the form of a Gorgoneion that could have marked the dedicat’s safe journey to this western, dangerous landmark and conjured her protection. That precise location of the item was within the large Gorham’s Cave, which has provided ample evidence of its spiritual significance to these mariners in the form of votive offerings. Gorham’s Cave itself was situated in a prominent location at the base of Calpe, the northern Pillar of Herakles, the marker of the end of the world beyond which lay the great and unknown ocean (Fig 4) [22]. The unique combination of myth, historical accounts, geography and archaeology leaves little doubt that for the ancient mariners of the Mediterranean the impressive geology and topography of Calpe, and within that Gorham’s Cave, had an importance that went beyond the purely practical, geographical and navigational. It seems that the key to the cave’s significance may have been the belief that it was the home of the Gorgons. Our observations on Calpe are in keeping with the religion of seafarers and worship in coastal caves in other parts of the Mediterranean, e.g. Malta or Ibiza [23–27].

## Supporting information

S1 File (DOCX)

S2 File Sites identified with Gorgoneia. (DOCX)
