# Correction: Where myth and archaeology meet: Discovering the Gorgon Medusa’s Lair

**DOI:** 10.1371/journal.pone.0250315

**Published:** 2021-04-13

**Authors:** The PLOS ONE Staff

There are errors in the author affiliations. The publisher apologizes for these errors. The correct affiliations are as follows:

Clive Finlayson^1,2,3,8^, Jose Maria Gutierrez Lopez^4^, M Cristina Reinoso del Rio^4^, Antonio M Saez Romero^5^, Francisco Giles Guzman^1^, Geraldine Finlayson^1,2,8*^, Francisco Giles Pacheco^1^, David Abulafia^6^, Stewart Finlayson^1,2,7^, Richard P Jennings^8^, Joaquin Rodriguez Vidal^9^

**1** The Gibraltar National Museum, Gibraltar, **2** The University of Gibraltar, Gibraltar Museum Campus, Gibraltar, **3** Department of Anthropology, University of Toronto, Canada, **4** Museo Histórico Municipal de Villamartín, Cádiz, Spain, **5** Department of Prehistory and Archaeology, University of Seville, Spain, **6** Gonville and Caius College, Cambridge University, United Kingdom, **7** Department of Life Sciences, Anglia Ruskin University, Cambridge, United Kingdom, **8** School of Biological and Environmental Sciences, Liverpool John Moores University, Liverpool, United Kingdom, **9** Department of Earth Sciences and CIPHCN-Heritage Centre, Campus del Carmen, University of Huelva, Huelva, Spain.
