# Supplementary material for: Where myth and archaeology meet: Discovering the Gorgon Medusa’s Lair
Source: PLoS One. 2021 Apr 1;16(4):e0249606. doi: 10.1371/journal.pone.0249606 (PMC8016213; doi:10.1371/journal.pone.0249606)
Supplement: S1 File — (DOCX) [file pone.0249606.s001.docx]

**SUPPLEMENTARY INFORMATION 1**

**Introduction**

The sanctuary at Gorham's Cave, located at the base of the European Pillar of Herakles, repeatedly referred to as Calpe by various ancient writers, is part of a network of caves that show evidence of use during the first millennium BCE and which punctuate the entire Mediterranean coast and the Atlantic coastline of the Iberian Peninsula^1^. The objects recovered from Gorham’s Cave are testimony of the accumulation of offerings and other ritual acts performed by sailors and other devotees who came to the sanctuary. Sanctuaries located in caves were linked to religious cults with strong esoteric components and eastern cultural character, which had oracular functions and acted as guarantors of maritime trade agreements through the protection granted by the deities that were represented in them^2^.

The archaeological evidence of the protohistoric occupation in Gorham’s Cave had not been investigated since the early 1970s^3^ and it was not until 1997 that the study of the interior section of Gorham’s Cave was integrated as part of the Gibraltar Caves Project led by the Gibraltar Museum^4^.

From an archaeological point of view, the key location of the sanctuary at the western end of the Mediterranean and at the entrance to the Atlantic Ocean, makes the site a magnificent laboratory from which to analyse the transit of goods that took place between both worlds, and establish who the participants in these voyages were in the general framework of the colonization of the west by different peoples.

**The Research to Date**

The current archaeological data suggest the beginning of the cultural use of Gorham’s Cave to the conventional archaic period (9^th^ – 6^th^ centuries BCE), established from a series of radiocarbon calibrated dates that fall between the end of 9^th^ century/beginning of 8^th^ century BCE (Beta 184053, 185544 and 185545)^5^. This period is marked by the presence of Phoenician offerings from the Near East, as well as amphorae and the typical red glazed ceramics produced in the first Phoenician colonies in the west, both on the Iberian Mediterranean coast and the newly founded western metropolis of Carthage^6^. Throughout the development of the archaic period of the sanctuary until the 6^th^ century BCE, Central Mediterranean artefacts of Sardinian and Nuragic origin, and ceramics from the local Tartessian area of the Guadalquivir^4^ have been identified. In the final moments of the archaic phase, in a period of clear hegemony of the ancient city founded by Tyre and the establishment of its successful fish salting economy, the products of the ancient extreme-western Phoenician colony of Cádiz appear^7^. It is the same period in which the presence of archaic Greek-oriental painted ceramics and Greek amphorae made in Ionia^4^, one of them with a graffito written in Phoenician^8,9^, can be confirmed in this area of the Strait of Gibraltar. This archaic period of the sanctuary is also highlighted by an important collection of scarabs in soapstone and faience of Egyptian manufacture^10^ and other offerings of different metals.

The archaeological context of the Classical period (5^th^ – 4^th^ centuries BCE) in this sanctuary reflects the intensification of the commercial activity in the area of the Pillars of Herakles. This eventually led to the consolidation of the cult in this sanctuary that had been initiated by the Mediterranean seafarers in the Archaic period. In this latter phase of use of the shrine, products of heterogeneous origins were again placed in the cave, but they corresponded to different areas of provenance than those detected for the Archaic period. Some of those areas were previously insignificant sources for the votives found at Gorham’s Cave, and others were not involved in cult activities during the Archaic phase. Among the latter would be the appearance of products from Ibiza, a Punic island city that played a key role as a redistribution centre for merchandise on the western side of the Mediterranean, of the North African Punic coastal cities, of the pre-Roman settlements of the south of Iberia, and also of the Greek regions, such as black-glazed Attic tableware vessels^4^. Equally noteworthy is the continuing constant arrival of Carthaginian items of the North African metropolis and of its Central Mediterranean sphere of influence, and Sardinian or Sicilian affiliations^6^. In this context, it is also necessary to highlight, for this phase, the use of scarabs made from green jasper, manufactured in the glyphic workshops of Sardinia^11^.

The later stages of the cave-sanctuary coincided with the Hellenistic phase (3^rd^ century 2^nd^ century BCE). They can be correlated with the progressive decline of the cult activities, as suggested by a far more limited and scarce archaeological record. Most of the offerings correspond to types which were quite characteristic throughout the western Mediterranean of that time, and that have been found in many diverse sites, both terrestrial and underwater, mostly dating to the 2nd century BCE^4^. These include a repertoire of goods that would be composed of the association of ceramic vessels of black-gloss Campanian A, Greco-Italic amphorae, plain and decorated pottery from the eastern Mediterranean coast of Iberia – even with the presence of a graffito in Greek-Iberian alphabet^8^ – as well as containers of perfumed Carthaginian oils. A range of evidence insinuate a connection between the end of the cult within Gorham’s Cave and the arrival of the Romans, and in particular with the process of colonization and Roman acculturation of the area of the Strait of Gibraltar. The cave’s abandonment as a sacred place probably was a consequence of the Roman annexation of the area following the defeat of Carthaginians in the Second Punic War of 206-202 BCE and the destruction of Carthage in 146 BCE^12^.

In short, the study of the archaeological material found in Gorham’s Cave reveals a growing wealth and variety corresponding to a long and complex history, built up by regular visits by people of very diverse types, origins and cultural backgrounds. This diversity turned the area into a centre whose liturgy and identity was forged over time, rather than through a continuous Phoenician cultural presence, as is usually assumed. In this regard, the special nature of a shrine such as that of Gorham’s Cave should also be taken into account. It should not necessarily be viewed in the context of a temple model characterized by regular worship led by a clergy that embodies it, and preserving the beliefs and practices, thus providing continuity. Instead, the special nature of this cave-sanctuary could have been such that both the practices and the beliefs were primarily those of the worshippers who, in turn, constituted the main character of the site^8,9^.

**Description of the object, ceramology, attribution, and workshop of origin of the terracotta with the Gorgoneion of Gibraltar.**

A set of ceramic fragments, with very specific and homogeneous morphological characteristics, have been isolated from the material found in the first level of the archaeological stratigraphy. These items have been brought to light over several archaeological seasons in Gorham’s Cave under the current Gibraltar Caves Project.

The positions where all the fragments have been recovered within the general planning grid of the cave (Q76, R80 and S79), allows us to reconstruct, with a good approximation, the original position where this offering was located in the context of the rest of the ritual deposits. Its situation in the deepest recesses at the back of the longitudinal gallery is remarkable, as the most preferential place from where it would be visible as one advanced into the cave.

A dozen fragments of the same non-vascular ceramic object have been identified, that is, not corresponding to a vessel or container. The reverse plane in all the fragments where this side has been preserved is unmistakable, without any marks of manufacture showing it to have been made with a potter's wheel. This suggests that it has been shaped as a plaque of fired earth. Before moving to the ceramic kiln, the piece was shaped by modelling the clay mass followed by the application of malleable clay to produce a detailed figurative representation in relief. Then the reverse was delicately smoothed and no evidence remains to indicate that the resulting surface was coloured with pigment. Due to the careful final treatment of the surfaces, the use of dies or partial moulds cannot be ruled out.

The ceramic clay body is red (MUNSELL 10R 5/8), compact and well decanted, and it can be observed that minerals of fine and medium size have been employed as degreasers. The composition is in the majority limestone and quartz nodules; in addition, the imprint corresponding to a bivalve microfossil can be observed. There is a presence of metamorphic rocks of the gneiss and mica-schist type along with traces of ferrous mineralization and grains of crushed ceramics. The clay was fired in an oxidizing atmosphere which was not completely regular as there is a section with a reddish tinge sandwiched between the lighter coloured outer layers representing different degrees of oxidation.

From the range of fragments which we have been describing, a partial reconstruction of the image represented on this ceramic plate has been possible as a number of these fragments fit with each other. In this way it has been possible to form an original continuous section of the piece that has a width of 160 mm. There are enough indicators among the conserved fragments for this refitted assemblage to be interpreted as a section of a figurative representation in high relief of part of a frontal view of a face which both eyes, parts of the nose and two segments of the eyebrows in the form of superciliary arches are preserved. There are several isolated fragments of the nose that retain the beginning of their attachment to the face and parts of its termination, reflected in the frontal perspective. Two very wide and open almond-shaped eyes stand out, in the interior of which there are two widely developed pupils formed by two discs in relief. The latter is a very peculiar feature among similar representations.

The piece recovered in Gorham's Cave, which to date remains incomplete, is an example of a well-known iconography used by Greek coroplasts of the archaic period^13^. The piece is a *Gorgoneion*, with missing fragments that would complete the image with open jaws bristling with teeth, a protruding tongue, and perhaps even some accompanied by serpents. It is a representation of the Gorgons, Medusa specifically, which according to the typology to which it has been ascribed, is of the ‘terrifying’ style^14^. We present the proposal for our reconstruction (Fig. 3), using as a base of contrast for the design of the image, a terracotta Antefix from Taranto conserved in the Archaeological Museum Città Metropolitana di Bari (Inventory Number 003094).

The Gorgoneion was quite a common decorative motif in the Greek world in different guises given its apotropaic power, because mythology gave the appearance of these monstrous figures a magical character, a lethal look that petrified their victim even after it was dead^15^. They were used abundantly as decoration in religious architecture from the beginnings of the temple model in the Greek world, making these images very visible by their use in these buildings^16,17,18^, with the intention of increasing the sense of mystery and awe that the faithful experienced before encountering the sacred^19^.

A review of the bibliography has been carried out and complemented with the consultation of the collections of different museums, in order to ascribe the correct chronology to the style of the Gorgoneion of Gorham’s Cave (Supplementary Information 2). This Gorgoneion is ascribed to the style of the period covering the entire sixth century BCE, but very likely the first half of the century. It appears distributed in a Greek cultural environment or its immediate surroundings, from Hellenic colonies in the Black Sea, Eastern Greece, Crete, the Peloponnese and continental Greece, Magna Graecia including Sicily and up to Etruria and Lazio. To this pattern of regional distribution, it would be necessary to add two outlying cases from the geographical and cultural point of view (Fig. 1). In the east Mediterranean there is a similar representation from Tell Dor, within a votive context dated in the last quarter of the sixth century, during the Persian rule^20^. The iconographic influence from the Greek world seems evident in the form, but within a culturally Semitic environment logic suggests a representation linked with minor deities of the Phoenician pantheon^21^. The other outlying case is located in the Balearic Islands, on the western end of the Mediterranean, in the ancient city of Ibiza, founded by the Phoenicians. This is an incomplete terracotta disk recovered in the great urban necropolis^22^, of more recent chronology (4th century BCE) but with a disputed iconographic attribution. Therefore, the example from Gorham's Cave becomes the *unicum* of the western boundary of the Mediterranean, in none other than the very Pillars of Herakles.

The Gorgoneion of Gibraltar requires a typological and formal definition. We have shown the extensive representation of the iconography of the Gorgon in the temple model of the Greek world, with a wide variety distributed mainly by their use in Greek architecture: architrave, entablature, cornices, pediments and ceilings^16,18^. Images of Gorgons can be seen in at least eight types of this architecture (Supplementary Information 2). These are: the akroterion, antefixes, apex antefixes, decorative plaques, the metopes, the pediment, revetment plaques, and the sima. It is important to note that the dimensions of the object from Gorham’s Cave exceed the most frequent measurement ranges for decorative elements such as antefixes, metopes, and sima. The preserved dimensions of the Gorgoneion of Gibraltar are far greater than those of any known antefix, which is why it is very unlikely that this is one. In addition, there is no trace of the half circular section with which an antefix is attached to the roof tiles. The measurements of the Gorgon of Gibraltar are more in line with decorative facing slabs and with the decoration of the pediments of temples such as those of Syracuse^23^.

However, consideration must be given to another interpretation: the possibility that this is an element of a mobile votive object of remarkable size. There are two options that could be considered: that of a portable ceramic altar, or perhaps more likely, that of a shield with a life size face of a Gorgon reproduced in terracotta, such as were made in workshops in the Greek area, e.g. Corinth. The option of whether it is a plaque from a temple (perhaps looted and finally deposited in Gibraltar) or simply manufactured and brought to Gorham's, is most likely, but other options are possible given that the piece it was not found in its original location and is incomplete. The piece could have been part of other types of supports also made in ceramic, such as small portable altars (e.g. SI 2, No. 20, from Kamarina, and other similar ones from Selinunte), or the votive shields produced in Corinth and other Greek workshops both on a reduced scale and at 1:1 size (e.g. SI 2, No. 73 and reference therein). Nevertheless, the alternative, that it was part of an altar, cannot be fully discarded. In any case, all the possibilities lead to the same conclusion: The Gorgoneion would have been in a very visible, prominent place, deep inside the cave, in an ideal position to be striking. In that location it could also have played a role in the activity of the main area of worship and deposition of offerings in the cave.

Regarding the description of the clay for the attribution of a workshop from which this votive offering from Gorham’s Cave originates, we are in a position to rule out that its characteristics suggest a Phoenician-Punic production with a Greek iconography as may have been initially supposed, since not even the ceramic clay of the object is consistent with an eastern Phoenician origin. The mineralogical characteristics of the Gorham’s Cave object have been compared visually with Magno-Greek, Sicilian^24^, and Corinthian productions, without being able to determine a specific origin, although this zone must be the area of provenance that has the greater arguments in its favour. There is archaeological evidence that representations of Gorgons with decorative function in architectural coatings were subject to transportation by sea, as discoveries underwater off the Sicilian coast suggest^25,26^. The Gorgoneion of Gibraltar travelled to the sanctuary at the extreme end of the world as did other goods produced in the Greek realm itself or the Magno-Greek, as supported by the underwater archaeological evidence in Iberia^27^, and arrived as votive offerings with the mariners of the end of the 7^th^ century and the all of the 6^th^ century.

**REFERENCES**

1 Aubet, Mª E. *Tiro y las colonias fenicias de Occidente*. 3ª Ed. Crítica, Barcelona (2009).

2 Bernardini, P. I Fenici ai confini del mondo: Le isole erranti e le Colonne di Melqart. *Sardinia, Corsica et Baleares Antiqvae. An International Journal of Archaeology* **I**, 111-121 (2003).

3 Culican, W. Phoenician remains from Gibraltar. *Australian Journal of Biblical Archaeology* **1**, 110-145 (1972).

4 Gutiérrez López, J. M., Reinoso del Rio, M. C., Giles Pacheco, F., Finlayson, C., Sáez Romero, A. M. La Cueva de Gorham (Gibraltar): Un Santuario Fenicio en El Confin Occidental del Mediterraneo. In Prados, F., Garcia, I., Bernard, G. (Eds.). *Confines. El Extremo del Mundo durante La Antiguedad.* Universidad de Alicante, Alicante (2012). pp 303-381.

5 Gutiérrez López, J. M., Reinoso del Rio, M. C., Giles Pacheco, F., Finlayson, C., Sáez Romero, A. M. Datos para la definición de la fase arcaica del santuario de la cueva de Gorham, Gibraltar**,** In A. Ferjaoui, T. Redissi (Eds.) *La Vie, la Mort et la Religion dans l’Univers Phénicien et Punique. Actes du VIIème congrès international des études phéniciennes et puniques.* Volume III. Tunis-Hammamet, Tunis (2019) pp 1783-1816.

6 Gutiérrez Lopez, J. M., Reinoso del Rio, M. C., Saez Romero, A. M., Giles Pacheco, F., Finlayson, C. J. La ofrendas de Hannon. El santuario de Gorham’s Cave (Gibraltar) y la navegacion cartaginesa atlantico-mediterranea. In Cocco, M. B., Gavini, A., Ibba, A. (Eds.). *L’Africa Romana XIX.* Estrato, Roma (2012) pp 3017-3032.

7 Sáez Romero, A. M., Higueras-Milena, A. The Phoenician and the Ocean: trade and worship at La Caleta, Cádiz, Spain. *The International Journal of Nautical Archaeology* **47 (1)**, 81-102 (2018).

8 Zamora Lopez, J. A., Gutierrez Lopez, J. M., Reinoso del Rio, M. C., Saez Romero, A. M., Giles Pacheco, F., Finlayson, J. C., Finlayson, G. Worship and cultures in Gorham’s Cave (Gibraltar): the sanctuary history and its written items. *Complutum* **214**, 113-130 (2013).

9 Gutiérrez López, J. M., Reinoso del Rio, M. C., Sáez Romero, A. M., Giles Pacheco, F., Finlayson, J. C., Zamora López, J. A. El Santuario de la Cueva de Gorham (Gibraltar). Estado de la cuestion. In Lemaire, A. (Ed.). *Pheniciens d’Orient et d’Occident.* Editions Jean Maisonneuve, Paris. (2014). pp 619-629.

10 Padró, J. *Egyptian-type documents from the mediterranean litoral of the Iberian Peninsula before the roman conquest. III. Study of the material, Andalusia*. Brill, Leiden (1985).

11 Guirguis, M., Enzo, S., Piga, G. Scarabei dalla necropoli fenicia e punica di Monte Sirai. Studio cronotipologico e archeometrico dei reperti rinvenuti tra il 2005 e il 2007. *International Journal of Archaeology* *Sardinia, Corsica et Baleares Antiqvae* **7**, 101-116 (2010).

12 Polybius. *The Rise of the Roman Empire*. Walbank, F. W. (Introd.), Scott-Kilvert, I. (Transl.). Penguin Books Limited, London (1980).

13 Krauskopf, I. Gorgo, gorgones. Gorgones (in Etruria). In *Lexicon Iconographicum Mythologiae Classicae* IV. Artemis, Zurich-Munich (1988).

14 Floren, J. Studien zur Typologie des Gorgoneion. Aschendorff, Münster (1977).

15 Wilk, S. R. *Medusa: solving the mystery of the Gorgon.* Oxford, Oxford University Press. (2000).

16 Belson, J. D. *The Gorgoneion in Greek Architecture*. Ph.D. dissertation, Bryn Mawr College, Pennsylvania (1981).

17 Winter, N. A. *Greek Architectural Terracottas: From the Prehistoric to the End of the Archaic Period*. Clarendon Press, Oxford (1993).

18 Danner, P. Westgriechische Giebeldekorationen, 1. Gorgoneia. *Romische Historische Mitteilungen* **42**, 19–105 (2000).

19 Marconi, C. *Temple Decoration and Cultural Identity in the Archaic Greek World: The Metopes of Selinus*. Cambridge Academic Press, New York. (2007).

20 Martin, R. S. From the East to Greece and back again: terracotta gorgon masks in a Phoenician context. In Lemaire, A. (Ed.). *Pheniciens d’Orient et d’Occident.* Editions Jean Maisonneuve, Paris. (2014). 289-299.

21 Culican, W. Phoenician Demons. *Journal of Near Eastern Studies* **35 (1)**, 21-24 (1976).

22 Almagro Gorbea, Mª J. *Corpus de las terracotas de Ibiza*. CSIC, Madrid (1980).

23 Benton, S. The Gorgon Plaque at Syracuse. *Papers of the British School at Rome* **22**, 132-137 (1954).

24 Barone, G., Mazzoleni, P., Raneri, S., Monterosso, G., Santostefano, A., Spagnolo, G., Vasta, V. Exploring the coroplasts’ ‘techne’ in greek architectural terracottas from Sicily: An archaeometric approach. Archaeometry 60-5, 986-1001 (2018).

25 Benneth, M., Paul, A. J. The Art of Magna Graecia. *Minerva. The International Review of Ancient Art and Archaeology* **13 (6)**, 9-16 (2002).

26 Fresina, A. Antefissa fittile con *gorgoneion*. In Agneto, F., Fresina, A., Oliveri, F., Sgroi, F., Tusa, S. (Eds.). *Mirabilia maris: tesori dai mari di Sicilia.* Editora Regione Siciliana, Palermo (2016). 252-253.

27 Santos Retolaza, M. Emporion, la nave de Cala Sant Vicenç y el comercio foceo en occidente a finales del siglo VI a. C. In Panvini, R., Guzzone, C., Sole, L. (eds.). *Traffici, commerci e vie di distribuzione nel Mediterraneo tra Protohistoria e V secolo a. C*. Editora Regione Siciliana, Palermo (2009). 243-254.
