# Supplementary material for: Where myth and archaeology meet: Discovering the Gorgon Medusa’s Lair
Source: PLoS One. 2021 Apr 1;16(4):e0249606. doi: 10.1371/journal.pone.0249606 (PMC8016213; doi:10.1371/journal.pone.0249606)
Supplement: S2 File — (DOCX) [file pone.0249606.s002.docx]

Supplementary Information 2. Sites identified with Gorgoneia

| **Nº** | **Site** | **Location** | **DESCRIPTION** | **ORIGIN** | **CHRONOLOGY BCE** | **REFERENCE** | **NOTES** |
| --- | --- | --- | --- | --- | --- | --- | --- |
| 1 | Gorham’s Cave | Gibraltar | clay plaque | cave sanctuary | VI | this paper |  |
| 2 | Puig des Molins, Ibiza | Balearic Islands | terracotta disc | Necropolis |  | Almagro Gorbea, 1980 | Museo Arqueológico Nacional nº 36189, Colección Vives y Escudero |
| 3 | Puig des Molins, Ibiza | Balearic Islands | terracotta disc | Necropolis |  | Costa & Fernández, 2003 |  |
| 4 | Akragas, Agrigento | Sicily | revetment plaques | east Olimpeion | first half VI | Belson, 1981 |  |
| 5 | Akragas, Agrigento | Sicily | moulds for relief | Pottery workshop | VI | Parello, 2014 |  |
| 6 | Gela | Sicily | antefix | Area Temple B |  | Lamagna, 2012 |  |
| 7 | Gela | Sicily | antefix | Building VI |  | Fiorentini, 1977 |  |
| 8 | Gela | Sicily | antefix | Acropolis | VI | B. Wohl, 1977 | Paul Getty Museum |
| 9 | Gela | Sicily | wall plaques | votive well in Athena Lindia temple | early VI | Belson, 1981 | Syracuse Museum |
| 10 | Gela, Bulala District | Sicily | antefix | underwater find | 2ª1/2 VI | Fresina, 2016 | VV.AA. Mirabilia Maris, 2016 |
| 11 | Megara Hyblaea, Augusta | Sicily | antefix |  | end VI | Pelagatti, 2006 |  |
| 12 | Echetla, Grammichele | Sicily | antefixes | unknown | end VI | Belson, 1981 | Syracuse Museum |
| 13 | Hybla Geleatis, Paterno | Sicily | antefix | necropolis area | late VI | Belson, 1981 | Antiquarium Paterno |
| 14 | Mendolito, Simeto | Sicily | antefix | oppidum | VI-V | Lamagna, 2012 |  |
| 15 | Randazzo | Sicily | antefix | unknown | VI | Lamagna, 2012 | Museo Archeologico Regionale “A. Salinas” di Palermo |
| 16 | Selinunte | Sicily | antefix | unknown |  | Lamagna, 2012 | Ex collezione Castelluccio. Museo archeologico di Palermo |
| 17 | Selinunte | Sicily | pedimental plaques | Temple C | middle VI | Belson, 1981 | Museo Archeologico di Palermo |
| 18 | Selinunte | Sicily | antefix | sanctuary Demeter Malophoros | late VI | Belson, 1981 | Museo Archeologico di Palermo |
| 19 | Selinunte | Sicily | antefix | unknown | late VI/early V | Robinson, 1923 | Royal Ontario Museum, Sturge collection |
| 20 | Kamarina | Sicily | terracotta little altar | unknown | fin VI-inicio V | Web Getty Images, Inc. | Museo Regionale di Kamarina |
| 21 | Kamarina | Sicily | antefix | unknown | late VI | Belson, 1981 | Museo Biscari, Catania |
| 22 | Monte Bubbonia, Mazzarino | Sicily | antefixes | archaic treasury, Akropolis | late VI-early V | Belson, 1981 | Syracuse Museum |
| 23 | Siracusa | Sicily | pedimental plaques | Athenaion | late VII/early VI | Benton, 1954; Mertens-Horn, 1995 | Syracuse Museum |
| 24 | Siracusa | Sicily | antefixes | unknown | late VI | Belson, 1981 | Syracuse Museum |
| 25 | Siracusa | Sicily | antefixes | Agora | late VI/early V | Belson, 1981 | Syracuse Museum |
| 26 | Morgantina, Serra Orlando | Sicily | antefixes | Farmhouse Hill Naiskos | VI-V | Kenfield, 1990 | first and second phase |
| 27 | Morgantina, Serra Orlando | Sicily | antefixes | Acropolis, Area III | second half VI | Belson, 1981 |  |
| 28 | Motya | Sicily | antefixes | unknown | late VI-early V | Belson, 1981 | Whitaker Museum, Motya |
| 29 | Motya | Sicily | terracotta disc | unknown | First half V | Poma 2016 | Whitaker Museum, Motya inv. 3113 |
| 30 | Naxos | Sicily | antefixes | Ship sheds (*Neoria*), port area | end VI/first quarter V | Lentini & Blackman, 2008; Lentini *et al*., 2008 |  |
| 31 | Naxos | Sicily | antefix | Tempietto H, Santa Venera | early VI | Lentini & Pakkanen, 2010 |  |
| 32 | Himera | Sicily | antefixes | Temple C | VI-V | Krauskopf, 1988 (LIMC), 293 | Antiquarium |
| 33 | Himera | Sicily | antefix | settlement | VI | Vassallo, 2015; 2016 |  |
| 34 | Cumae | Campania, Italy | clay relief |  | mid VI | Krauskopf, 1988 (LIMC), 291 | National Museum Naples |
| 35 | Taranto | Magna Graecia | antefixes |  | second half VI-begin V | Krauskopf, 1988 (LIMC), 293-294 |  |
| 36 | Taranto | Magna Graecia | antefixes |  | mid V | Krauskopf, 1988 (LIMC), 295 |  |
| 37 | Taranto | Magna Graecia | antefix | unknown | VI-V ? | communication with Museum | Museo Archeologico di Santa Scolastica, Bari. Inventory nº 3094 |
| 38 | Arpi | Puglia, Italy | antefix | unknown | V | Todisco, 1992 | Museo Cívico Foggia |
| 39 | Capua | Campania, Italy | antefixes |  | second half VI-first quarter V | Krauskopf, 1988 (LIMC), 294 | Museo Archeologico dell’Antica Capua |
| 40 | Capua | Campania, Italy | *sima* decoration |  | second half VI | Rescigno & Sampaolo, 2010 | Museo Provinciale Campano- Fondo Patturelli |
| 41 | Capua Vetere, Santa Maria | Campania, Italy | terracotta revetments plaques | burial goods | mid-V | Gilotta, 2007 | Museo Archeologico dell’Antica Capua |
| 42 | Locri Epizefiri | Calabria, Italy | antefixes | ‘stoà a U', Persephone shrine, Centocamere | end VII/ first half V | Grillo, 2010 |  |
| 43 | Crotone | Calabria, Italy | antefixes | several provenances | end VI | Aversa, 2010 |  |
| 44 | Caulonia, San Marco | Calabria, Italy | antefix | domestic context | end VI | Lepore, 2010 |  |
| 45 | Caulonia, San Marco | Calabria, Italy | antefixes | Doric temple, Punta Stilo | late VI | Belson, 1981 | Reggio Calabria Museum |
| 46 | Krimisa, Cirò Marina | Calabria, Italy | antefixes | Apollo Sanctuary, Punta Alice | mid-V | Belson, 1981 | Reggio Calabria Museum |
| 47 | Hipponion, Vibo Valentia | Calabria, Italy | revetment plaque | unknown | late VI/early V | Belson, 1981 | Reggio Calabria Museum |
| 48 | Hipponion, Vibo Valentia | Calabria, Italy | antefixes | unknown | late VI/early V | Belson, 1981 | Reggio Calabria Museum |
| 49 | Sibari, Cosenza | Calabria, Italy | antefix | Casa Bianca area, season 2012 | mid-VI | Greco, 2014 |  |
| 50 | Monte Sannace, Gioia Del Colle, Bari | Puglia, Italy | antefix | megaron, G3 area | second half VI | Galeandro & Palmentola, 2013 |  |
| 51 | Torre Di Satriano, Potenza | Basilicata, Italy | terracotta revetments plaque | *anaktoron* | mid-VI | Capozzoli & Osanna, 2009 |  |
| 52 | Serra Di Vaglio, Potenza | Basilicata, Italy | antefixes | settlement | V | Greco, 1982 |  |
| 53 | Metapontion, Matera | Basilicata, Italy | antefixes | Temple D | early V | Belson, 1981 |  |
| 54 | Metapontion, Matera | Basilicata, Italy | revetment plaque | unknown | late VI/early V | Belson, 1981 | Antiquarium Metaponto |
| 55 | Poseidonia, Paestum | Salerno, Italy | antefixes | unknown | late VI | Belson, 1981 | Paestum Museum |
| 56 | Poseidonia, Paestum | Salerno, Italy | antefix | Temple of Hera II | first half V | Belson, 1981 | Paestum Museum |
| 57 | Vignanello | Lazio, Italy | antefix |  | first half VI | Krauskopf, 1988 (LIMC), 331 | Villa Giulia Museum |
| 58 | Satricum | Borgo Le Ferriere, Lazio | antefix | temple dedicated to Mater Matuta | mid VI | Krauskopf, 1988 (LIMC), 332 |  |
| 59 | Roman Forum | Lazio, Italy | antefixes | Regia, third phase | first half VI | Winter, 2017 |  |
| 60 | Sant'Omobono, Rome | Lazio, Italy | terracotta slab | Temple of Mater Matuta, triangular pediment | first half VI | Winter, 2017 |  |
| 61 | Pyrgi, C. Santa Severa, Sta. Marinella | Lazio, Italy | terracotta relief | Temple A | mid V | Krauskopf, 1976 | Villa Giulia Museum, Roma |
| 62 | Poggio Civitate, Murlo | Etruria, Italy | antefixes |  | first half VI | Krauskopf, 1988 (LIMC), 331; Neils, 1976 |  |
| 63 | Veio, Portonaccio | Etruria, Italy | antefix | Minerva shrine | end VI | Krauskopf, 1988 (LIMC), 332 | Villa Giulia Museum |
| 64 | Falerii | Etruria, Italy | akroter | Lo Scasato | end IV/early III | Krauskopf, 1988 (LIMC), 333 | Villa Giulia Museum |
| 65 | Orvieto | Umbria, Italy | akroter | Belvedere temple | IV | Krauskopf, 1988 (LIMC), 333 | C. Faina Museum |
| 66 | Nauplion | Argolid, Greece | round-shaped clay masks |  | VII | Krauskopf, 1988 (LIMC), 289 |  |
| 67 | Athens | Greece | antefixes | Agora | mid VI | Nicholis, 1970; Krauskopf, 1988(LIMC), 291 |  |
| 68 | Athens | Greece | antefixes | Acropolis | end VI | Vlassopoulou y Touloupa, 1990 | Acropolis Museum |
| 69 | Spata (Attica) | Greece | antefix mould | unknown | mid V | Krauskopf, 1988 (LIMC), 295 | British Museum |
| 70 | Olympia | Greece | *sima* decoration |  | mid V | Krauskopf, 1988 (LIMC), 295 | Olympia museum |
| 71 | Olympia | Greece | antefix | Bouleuterion | first half VI | Heiden, 1990 |  |
| 72 | Corinth | Greece | terracotta reliefs | The Potters' Quarter | first quarter VI | Stillwell, 1952 |  |
| 73 | Corinth | Greece | small votive shield | The Potters' Quarter | second half VI | Stillwell, 1952 |  |
| 74 | Corinth | Greece | mould | The Potters' Quarter | IV | Stillwell, 1948 |  |
| 75 | Corinth | Greece | terracotta plaque | Asklepieion | VI-IV | Roebuck, 1951 |  |
| 76 | Kalydon, Etoloakarnania | Continental Greece | terracotta fragments | sanctuary of Artemis Laphria | first quarter VI | Dyggve, 1948 |  |
| 77 | Antikyra | Boeotia, Greece | antefixes | Archaic temple, ¿*Athenaion*? | first half VI | Belson, 1981; Krauskopf, 1988 (LIMC), 292 |  |
| 78 | Ptoion | Boeotia, Greece | antefix | unknown | second half VI | Belson, 1981 |  |
| 79 | Mantineia, Paleópoli | Arcadia, Greece | antefix | unknown | V | Belson, 1981 | National Museum, Athens |
| 80 | Thermon, Etolo-Akarnania | Greek Mainland | terracotta metope | temple Apollo Thermios | second half VII | Belson, 1981 |  |
| 81 | Palaikastro, Heleia | Crete | antefixes | temple Diktaian Zeus | late VI/early V | Belson, 1981 | Heraklion Museum |
| 82 | Praisos | Crete | antefix | temple Diktaian Zeus | late VI/early V | Belson, 1981 |  |
| 83 | Mon Repos, Corfu | Ionian Islands, Greece | antefixes, plaques | Heraion | end VII | Sapirstein, 2012 |  |
| 84 | Didyma | Eastern Aegean, Turkey | antefixes | Sacred Way, temenos | second half VI | Schneider, 1990 |  |
| 85 | Cebren | Troad, Eastern Aegean, Turkey | antefixes | unknown | second half VI | Belson, 1981 |  |
| 86 | Klazomenai | Eastern Aegean, Turkey | antefixes | unknown | second half VI | Belson, 1981 | Prague Museum |
| 87 | Larisa on the Hermos | Eastern Aegean, Turkey | *sima* decoration | small sanctuary | second half VI | Belson, 1981 |  |
| 88 | Lesvos, Antissa | Eastern Aegean, Greece | antefix | unknown | end VI | Belson, 1981 |  |
| 89 | Samos | Eastern Aegean, Greece | antefixes | Heraion | end VI | Ohnesorg, 1990 |  |
| 90 | Miletos, Kalabaktepe | Eastern Aegean, Turkey | antefixes |  | end VI | Schneider, 1990 |  |
| 91 | Thasos | Northern Aegean, Greece | antefix | Herakles temple y Prytaneion | second half VI | Krauskopf, 1988 (LIMC), 292 |  |
| 92 | Thasos | Northern Aegean, Greece | antefixes | *Prytaneion* | VI-V | Picard & Avezou, 1914; Winter, 1990 | Thasos Museum |
| 93 | Histria | Istros, Romania | antefixes | temples zone | second half VI | Zimmermann, 1990 |  |
| 94 | Tel Dor | Israel | terracotta mask ? | Area D2. Deposit B. Cultic deposit | 4º 1/4 VI, Persian period | Martin, 2014; Nitschke et al., 2011 |  |
| 95 | Tel Dor | Israel | terracotta mask ? | Area D2. Deposit A. | Persian period | Martin, 2014 | Fragment |

**References**

Almagro Gorbea, Mª J. *Corpus de las terracotas de Ibiza*. (CSIC, 1980).

Aversa, G. Produzioni di coroplastica architettonica tra Crotone e Kaulonia: elementi di raffronto e spunti di riflessione. In *Caulonia tra Crotone e Locri* (eds L. Lepore & P. Turi) 199-207, <https://doi.org/10.1400/148386> (Firenze University Press, 2010).

Belson, J. D. *The Gorgoneion in Greek Architecture*. <http://repository.brynmawr.edu/dissertations/142> (Bryn Mawr College, 1981).

Benton, S. The Gorgon Plaque at Syracuse. *Papers of the British School at Rome* **22**, 132-137, <http://www.jstor.org/stable/40310519> (1954).

Capozzoli, V. & Osanna, M. Da Taranto alla *mesogaia* nord-lucana: le terrecotte architettoniche dell’*anaktoron* di Torre di Satriano. *Ostraka. Rivista di Antichità* **18**, 141-174 (2009).

Costa, B. & Fernández, J.H. El rostro de la muerte: representaciones de gorgoneia en la

necrópolis del Puig des Molins. In B. Costa, J.H. Fernández (Eds.) *Misceláneas de arqueología ebusitana,* II *El Puig des Molins (Eivissa): un siglo de investigaciones*. Ibiza (2003), pp 197-250.

Fiorentini, G. Sacelli sull’acropoli di Gela e a Monte Adranone nella Valle del Belice. *Cronache di Archeologia* **16**, 105-114 (1977).

Fresina, A. Antefissa fittile con gorgoneion. In *Mirabilia maris: tesori dai mari di Sicilia* (eds F. Agneto, A. Fresina, F. Oliveri, F. Sgroi & S. Tusa) 252-253 (Regione Siciliana, 2016).

Galeandro, F. & Palmentola, P. Gli scavi della Scuola di Specializzazione in Beni Archeologici dell’Università di Bari sull’acropoli di Monte Sannace (1994-2001). In *Epigrafia e territorio, politica e società: Temi di Antichità Romane*, IX (eds M. **Chelotti & M. Silvestrini)** 31-110 (Edipuglia, 2013).

Greco, G. Lo sviluppo di Serra di Vaglio nel V e IV sec. a. C. *Mélanges de l'Ecole Française de Rome. Antiquité* **94**, 67-89, https://doi.org/10.3406/mefr.1982.1316 (1982).

Greco, E. Un’antefissa arcaica da Sibari. In *Miti e popoli del Mediterraneo antico. Scritti in onore di Gabriella d’Henry* (eds C. Lambert & F. Pastore) 167-172 (Arci Postiglione, 2014).

Gilotta, F. Zeitstil e meccanismi di trasmissione nella piccola plastica decorativa capuana di epoca tardo-arcaica. *Orizzonti. Rassegna di Archeologia* **7**, 49-80, https://doi.org/ [10.1400/81985](http://dx.medra.org/10.1400/81985) (2007).

Grillo, E. Antefisse con *Gorgóneion* tra Locri e Caulonia. In *Caulonia tra Crotone e Locri.* (eds L. Lepore & P. Turi) 363-374 (Firenze University Press, 2010).

Heiden, J. Die Archaischen Dächer von Olympia. *Hesperia: The Journal of the American School of Classical Studies at Athens* **59**, 41-46, <http://www.jstor.org/stable/148123> (1990).

Kenfield, J. F. An East Greek master coroplast at late archaic Morgantina. *Hesperia: The Journal of the American School of Classical Studies at Athens* **59**, 265-274. <http://www.jstor.org/stable/148141> (1990).

Krauskopf, I. Gorgonein darstellungen auf etruskischen münzen und in der etruskischen Kunst. In *Contributi introduttivi allo studio della monetazione etrusca. Atti del V Convegno Internazionale di Studi Numismatici.* 319-348 (Istituto Italiano di Numismatica, 1976).

Krauskopf, I. Gorgo, gorgones. In Lexicon Iconographicum Mythologiae Classicae IV. 285–330 (Artemis, 1988).

Krauskopf, I. Gorgones (in Etruria). In Lexicon Iconographicum Mythologiae Classicae IV. 330-345 (Artemis, 1988).

Lamagna, G. Gorgoneia arcaici dalla Sicilia orientale*.* In *La Sicilia in Età Arcaica.* *Dalle apoikiai al 480 a.C.* (eds R. Panvini & L. Sole) 231-236 (Salvatore Sciascia Editore, 2012).

Lentini, M. C. & Blackman, D. J. I *neoria* di Naxos in Sicilia. *Archeologia Classica* **59**, 1-38, <http://www.jstor.org/stable/44367725> (2008).

Lentini, M. C.; Blackman, D. J. & Pakkanen, J. The shipsheds of sicilian Naxos: a second preliminary report (2003–6). *Annual of the British School at Athens* **103**, 299-366, <https://doi.org/10.1017/S0068245400000101> (2008).

Lentini, M. C. & Pakkanen, J. The sanctuary to the west of the Santa Venera: a review. In *Deliciae Fictiles IV. Architectural Terracottas in Ancient Italy. Images of Gods, Monsters and Heroes* (eds P. Lulof & C. Rescigno) 417-425 (Oxbow Books, 2010).

Lepore, L. Gli scavi in località S. Marco nord-est: dall’*oikos* arcaico alla sistemazione ellenistica. In *Caulonia tra Crotone e Locri* (eds L. Lepore & P. Turi) 81-113 (Firenze University Press, 2010) <https://doi.org/10.1400/148377>

Marconi, C. The Greek West: Temples and their Decoration. In *A Companion to Greek Architecture* (ed M. M. Miles) 75-91 (John Wiley & Sons, 2016).

Martín, S. R. From the East to Greece and back again: Terracotta gorgon masks in a phoenician

context. In *Phéniciens d’Orient et d’Occident. Mélanges Josette Elayi* (ed A. Lemaire) 289-299 (Editions Jean Maisonneuve, 2014).

Neils, J. The terracotta gorgoneia of Poggio Civitate (Murlo). *Römische Mitteilungen* **83**, 1-29 (1976).

Nicholls, R. Architectural Terracotta Sculpture from the Athenian Agora. *Hesperia: The Journal of the American School of Classical Studies at Athens* **39**, 115-138. <http://www.jstor.org/stable/147450> (1970).

Nitschke, J. L.; Martin, S. R. & Shalev, Y. Between Carmel and the Sea. Tel Dor: The Late Periods. *Near Eastern Archaeology* **74:3**, 132-154 (2011).

Ohnesorg, A. Archaic Roof Tiles from the Heraion on Samos. *Hesperia: The Journal of the American School of Classical Studies at Athens* **59**, 181-192. <http://www.jstor.org/stable/148133> (1990).

Parello, M. C. Gli ergasteria di Akragas: nuove piste di ricerca. In *Le opere e i giorni: lavoro, produzione e commercio tra passato e presente* (ed V. Caminneci) 181-202 (Soprintendenza BB.CC.AA. di Agrigento, 2014).

Picard, Ch. & Avezou, Ch. Les fouilles de Thasos (1913). *Comptes Rendus des Séances de l'Académie des Inscriptions et Belles-Lettres* **58**, 276-305, <http://www.persee.fr/doc/crai_0065-0536_1914_num_58_3_73402> (1914).

# Pelagatti, P. Tipi inediti o rari di antefisse archaiche tra Sicilia e Magna Grecia. Soggetti i culti. In *Deliciae Fictiles III. Architectural Terracottas in Ancient Italy. New Discoveries and Interpretations* (eds I. Edlund-Berry, G. Greco & J. Kenfield) 433-451 (Oxbow Books, 2006).

Poma, L. Alcuni temi iconografici greci sugli « stampi per focaccia » punici. *Rivista di Studi Fenici* **XLIV**, 219-232.

Rescigno, C. & Sampaolo, V. La decorazione del vano e del fastigio frontonale tra Cuma e Capua. In *Deliciae Fictiles IV. Architectural Terracottas in Ancient Italy. Images of Gods, Monsters and Heroes* (eds P. Lulof & C. Rescigno) 296-318 (Oxbow Books, 2010).

Robinson, D. M. Etruscan-Campanian Antefixes and Other Terra-Cottas from Italy at the Johns Hopkins University. American Journal of Archaeology **27**, 1-22, <http://www.jstor.org/stable/497529> (1923).

Roebuck, C. *Corinth XIV. The Asklepieion and Lerna. Based on the excavations and preliminary studies of F. J. de Waele* (American School of Classical Studies at Athens, 1951).

Sapirstein, Ph. The Monumental Archaic Roof of the Temple of Hera at Mon Repos, Corfu. *Hesperia: The Journal of the American School of Classical Studies at Athens* **81**, 31-91. <http://www.jstor.org/stable/10.2972/hesperia.81.1.0031> (2012).

Schneider, P. New Information from the Discovery of an Archaic Tiled Roof in Ionia. *Hesperia: The Journal of the American School of Classical Studies at Athens* **59**, 211-222. <http://www.jstor.org/stable/148136> (1990).

Stillwell, A. N. *Corinth XV-1. The Potters' Quarter* (American School of Classical Studies at Athens, 1948).

Stillwell, A. N. *Corinth XV-2. The Potters' Quarter*. *The terracottas* (American School of Classical Studies at Athens, 1952).

Todisco, L. Coroplastica. In *Introduzione all’artiagianato della Puglia antica dall’ettà coloniale dall’ettà romana* (eds L. Todisco, G. Volpe, A. Bottini, P. G. Guzzo, F. F. Troisi & M. Chelotti) 71-86 (Edipuglia, 1992).

Vassallo, S. Il contributo delle importazioni allo sviluppo e all’identità culturale di Himera. In *Materielle Kultur und Identität im Spannungsfeld zwischen mediterraner Welt und Mitteleuropa* (ed H. Baitinger) 69-79 (Römisch-Germanisches Zentralmuseum, 2016).

Vlassopoulou, Ch. & Touloupa, E. Decorated Architectural Terracottas from the Athenian Acropolis: Catalogue of Exhibition. *Hesperia: The Journal of the American School of Classical Studies at Athens* **59**, pp. i-xxxi, <http://www.jstor.org/stable/148146> (1990).

Winter, N. A. Traders and Refugees: Contributions to Etruscan Architecture. *Etruscan Studies* **20**, 123–151, <https://doi.org/10.1515/etst-2017-0016> (2017).

Wohl, B. A gorgon antefix from Gela in the J. P. Getty Museum. *The J. Paul Getty Museum Journal* **5**, 75-78, <http://www.jstor.org/stable/4166367> (1978).

Zimmermann, K. Archaische Dachterrakotten aus Histria. *Hesperia: The Journal of the American School of Classical Studies at Athens* **59**, 223-233, <http://www.jstor.org/stable/148137> (1990).
